# Cohort profile: The FarmMERGE project—Merging human and animal databases to investigate the relationship between farmer and livestock health and welfare. The HUNT Study

**DOI:** 10.1371/journal.pone.0301045

**Published:** 2024-03-28

**Authors:** Magnhild Oust Torske, Natalie Steen, Jonil Tau Ursin, Steinar Krokstad, Håvard Nørstebø, Karianne Muri

**Affiliations:** 1 Faculty of Biosciences and Aquaculture, Nord University, Steinkjer, Norway; 2 Faculty of Biosciences and Aquaculture, Nord University, Bodø, Norway; 3 HUNT Research Centre, Department of Public Health and Nursing, Faculty of Medicine and Health Sciences, Norwegian University of Science and Technology (NTNU), Levanger, Norway; 4 Levanger Hospital, Nord-Trøndelag Hospital Trust, Levanger, Norway; 5 TINE SA, Farm Advisory Services, Research and & Development Department, Ås, Norway; 6 Department of Production Animal Clinical Sciences, Faculty of Veterinary Medicine, Norwegian University of Life Sciences (NMBU), Ås, Norway; Wroclaw University of Environmental and Life Sciences: Uniwersytet Przyrodniczy we Wroclawiu, POLAND

## Abstract

Stockmanship is an important determinant for good animal welfare and health. The goal of the FarmMERGE project is to investigate the associations between farmer health and work environment, and the health, productivity and welfare of their livestock. We merged several livestock industry databases with a major total population-based health study in Norway (The Trøndelag Health Study 2017–2019 (HUNT4)). This paper describes the project’s collection and merging of data, and the cohort of farmers and farms that were identified as a result of our registry merge. There were 56,042 participants of HUNT4 (Nord-Trøndelag County participants only, participation rate: 54.0%). We merged a list of HUNT4 participants whose self-reported main occupation was “farmer” (n = 2,407) with agricultural databases containing production and health data from sheep, swine, dairy and beef cattle from 2017–2020. The Central Coordinating Register for Legal Entities was used as an intermediary step to achieve a link between the farmer and farming enterprise data. We identified 816 farmers (89.5% male, mean age 51.3 years) who had roles in 771 farming enterprises with documented animal production. The cohort included 675 unique farmer-farm combinations in cattle production, 139 in sheep, and 125 in swine. We linked at least one HUNT4 participant to approximately 63% of the dairy farms, 53% of the beef cattle farms, 30% of the sheep farms, and 38% of the swine farms in Nord-Trøndelag County in the 2017–2019 period. Using existing databases may be an efficient way of collecting large amounts of data for research, and using total population-based human health surveys may decrease response bias. However, the quality of the resulting research data will depend on the quality of the databases used, and thorough knowledge of the databases is required.

## Introduction

Stockmanship is acknowledged as the single most important influence on farm animal welfare [1], and therefore a topic of great relevance in animal welfare research. Animal welfare is multifactorial and commonly seen as a combination of the animals’ basic health and functioning, their ability to carry out natural behaviors with access to natural elements in their environment, as well as their affective state [2]. Consequently, animal welfare can be affected by several factors related to the animals’ physical and social environment. This includes the environment they are kept in, the animal-animal interactions, and the stockperson’s handling and management of the flock.

Good stockmanship requires skillful and sympathetic animal handling; early recognition of pain, sickness or injury; the provision of appropriate care; as well as a production system that encourages the development of competence and empathy [3]. However, if the wellbeing of a stockperson is poor, or the farmers’ functional level is reduced due to illness, it may reduce his or her ability to ensure acceptable standards of animal welfare [4].

Farming has been described as a “*high stress and dangerous occupation*” [5 p.346]. The work environment of farmers is often characterised by a high workload, long working hours, solitary work, heavy physical labor and a poor work-life balance [6, 7]. Research has shown that farmers in several countries experience a variety of health problems, often with epidemiological characteristics that differ from other occupations [8–10]. Working farmers have been found to be disproportionately affected by occupational injuries [11, 12], chronic musculoskeletal problems [13, 14], chronic respiratory conditions [15–18], depression, anxiety and suicide [19–24], and a general reduced quality of life in older age [25]. The gender and age demographics of farming populations, as well as socio-cultural characteristics, are amongst possible factors being explored [26, 27].

The physical work environment of farmers is highly dependent on the state of the farm buildings and equipment available, as well as the type of farming. Conditions of the physical work environment can directly impact on the health and welfare of both the animals and the farmer, in addition to influencing the human-animal relationship [28]. Sheep farmers with a higher perceived exposure to physical burdens in their work environment have been shown to have a lower level of job satisfaction [29]. Furthermore, the construction year of the farm building has been linked to job satisfaction; the newer the building, the higher the job satisfaction among dairy farmers [30]. Recently it has been shown that high occupational well-being and low levels of stress in dairy farmers are positively associated with animal welfare [31]. However, results are conflicting—a Swedish study found that when farmers had a higher load of physical symptoms that indicated illness or injury, the disease rates in their cattle were lower [32].

The role stockpeople have as professional managers of animals, and how they thus are fundamental in safeguarding farm animal welfare, is often not given proper appreciation, even by farmers themselves [33]. As farmer wellbeing and livestock welfare are likely to be intertwined, the UK Farm Animal Welfare Committee (FAWC) has pointed out that there is a need for increased awareness and recognition of farmers’ wellbeing, and how this may influence farm animal welfare [4]. Thus, research on the connections between stockperson health and wellbeing and farm animal welfare is essential to contribute to improvement of farmers’ suboptimal social, economic and health situation. There are several ways of obtaining data for research on the human-animal relationship and stockmanship, including experiments, observational studies, interviews, questionnaires, and using pre-existing data. In this study, we have performed an innovative and complex merging of several pre-existing human and livestock databases in order to learn more about the interrelationships between human wellbeing and animal welfare in livestock farming. To our knowledge, the merging of data from human and animal databases at this scale has not previously been performed in Norway. The goal of this paper is therefore to describe this data merging process, and to provide a cohort profile of the resulting cohort of farmers and their farms.

## Cohort description

### Study design and source population

The FarmMERGE project relies on a complex merging of registry data from three different sectors; one total population-based health study, several agricultural industry registries with livestock data, and one official registry of all organizations and enterprises in Norway. The latter was a necessary intermediate step to enable merging of human and livestock data.

The data in our study comes from Nord-Trøndelag County, which is one of the main agricultural areas of Norway. Norwegian farming is characterized by individual, self-owning, independent farmers [34], whose farms are usually fairly small compared to many other industrialized countries [35]. On most farms, the farmer-operator and their partner does most of the farmwork, with limited use of hired farmworkers [36]. Dairy is the largest production form in Nord-Trøndelag County (47% of the gross agricultural production in 2015), but there is also a substantial production of poultry (broilers and egg production), pork, sheep, beef, and crops (e.g., vegetables, barley and other cereals) [37].

In 2018, the counties of Nord-Trøndelag and Sør-Trøndelag were formally merged into the new administrative unit of Trøndelag County [38]. In 2017, the last year for which there is separate data for Nord-Trøndelag County available, there were 2,016 agricultural enterprises with livestock in Nord-Trøndelag County [39]. A full list of agricultural enterprises broken down by animal production is available in S1 Table. In general, farms in Nord-Trøndelag County are somewhat larger than the Norwegian average [40–42], as shown in S2 Table.

### Human databases

**The Trøndelag Health Study** (the HUNT Study) is one of the most comprehensive population-based health surveys in the world, with four data collections over the course of four decades (HUNT1 in 1984–86, HUNT2 in 1995–97, HUNT3 in 2006–2008 and HUNT4 in 2017–2019) [43–45]. In this study, we used data from the HUNT4 Survey. The data collection took place between August 29th, 2017 and February 23rd, 2019. We only used participants from the northern part of the County (Nord-Trøndelag), as occupational information was not known for participants from the southern part of the county. In total, 56,042 residents of Nord-Trøndelag County participated in HUNT4 (response rate: 54.0%) [45].

The HUNT4 data collection was extensive and included a range of questionnaires, an interview, clinical measurements, and biological samples. All residents (103,800) of Nord-Trøndelag County aged 20 and above were invited to participate [45]. Invitations were sent by post to the address registered in the National Population Register [46]. The envelope contained an information form and Questionnaire 1 (Q1). Q1 could either be completed and returned online (approximately 30% of respondents) or delivered physically at the field stations (approximately 70% of the respondents) (J. S. Fenstad, personal communication, December 5, 2022). The in-person part of the data collection was performed at a mobile field station, where the participants had an interview, undertook clinical examinations, had biological samples taken, and were given a second questionnaire (Q2) to complete. More information about the HUNT4 Survey, including a cohort profile, is available elsewhere [45]. The HUNT4 data was first made available to the researchers on March 8th, 2022.

**The Central Coordinating Register for Legal Entities** (CCR) [47] is managed by the Brønnøysund Register Center [48]. The CCR collects basic data about enterprises in Norway, e.g., business name, type of enterprise, activities, and the identity of people with key roles (e.g., owner, board member) in the enterprise. The CCR assigns a nine-digit **organization number** to all registered enterprises, which is also used as an identifier in other registries [47].

### Livestock databases

Several organizations associated with Norwegian agriculture own and maintain databases that contain a range of livestock health, welfare, and production data. Some databases are membership-based, hence only subsets of the farms are included in these databases, whereas other databases contain data routinely collected from all Norwegian farms.

#### Non-membership-based livestock databases

**Slaughter data** is collected at all abattoirs throughout the country. The data includes carcass weights and classifications [49], disease codes registered at the meat inspection [50], and registration of various animal welfare indicators (e.g., tail biting and dirty animals sent to slaughter) [50, 51].

**Veterinary treatment data** originated from the Animal Health Recording System (*Dyrehelseportalen)* [52]. According to §17 in the Norwegian Act relating to Veterinarians and Other Animal Health Personnel, all treatments of terrestrial animals with prescription drugs must be initiated by a licensed veterinarian [53]. Farmers are also required to record treatments and diseases of their animals [54].

#### Membership-based livestock databases

The **Norwegian Dairy Herd Recording System** (*Kukontrollen*, NHRS-Dairy) [55] is a dairy cow health and production database. NHRS-Dairy incorporates data from a number of sources, including animal health registrations by veterinarians, dairies, and abattoirs, as well as registrations made by the farmers themselves. Approximately 97% of Norwegian dairy farmers are members of NHRS-Dairy [55]. More information about the NHRS-Dairy and its validity in epidemiological research is available elsewhere [56, 57].

The **Norwegian Beef Herd Recording System** (*Storfekjøttkontrollen*, NHRS-Beef) is an animal health and production registry for beef cattle [58].

The **Norwegian Sheep Herd Recording System** (*Sauekontrollen*, NHRS-Sheep) is an animal health and production registry for sheep meat production [59].

The **Norwegian Swine Herd Recording System** (*Ingris*, NHRS-Swine) is an animal health and production registry for pork production [60].

The membership rates of the Herd Recording systems for beef cattle, sheep and swine vary considerably between production forms, geographical areas and over time; but overall they are considerably lower than the NHRS-Dairy membership rates, ranging from 12.7% (fattening pigs) to 69% (beef cattle) in 2018 [61–63].

Based on an expected HUNT4 response rate among farmers similar to that of the general population (approximately 50%) [45], we considered the number of farming enterprises with dairy goats, egg and broiler production too low to be used in statistical analyses (S1 Table). Consequently, we did not proceed with any data merging in these species.

### Selection of study participants from HUNT4

There were 56,042 persons who participated in HUNT4 (returned Q1), 53,806 participated in the field station interview, and 42,763 returned Q2 [45]. Registration of occupation in HUNT4 was done during the field station interview, in which participants were asked the question: *“What is/was the name of your main occupation*?*”* Only participants who stated that they were currently or previously occupationally active were asked this question. The reply was classified by the interviewer according to the *Standard for Yrkesklassifisering* (STYRK 98), which is based on the International Standard Classification of Occupations (ISCO-88(COM)) [64, 65]. Occupations are divided into 10 broad groups (numbered from 0 to 10), with farmers belonging to group 6: “*Skilled agricultural and fishery workers”*. Each occupational group is further divided into subgroups, with up to four levels. In HUNT4, participants were classified on all four levels, but only the first three occupational levels are available to researchers to protect the anonymity of study participants.

After excluding those with no reported current or previous occupation (n = 13,129), occupation code “military” (n = 91) or “Non-specified occupation” (n = 561), there were 40,025 remaining participants. The STYRK code that was used to identify farmers in our study, was “*61 Agricultural workers*” (n = 2,407). Its more specific subcodes include: “*611 Market gardeners and crop growers*” (n = 214), “*612 Animal Producers*” (n = 1,295) and “*613 Crop and animal producers*” (n = 887). Eleven of the participants identified as *“61 Agricultural workers”* did not have a more specific three-digit code. “*611 Market gardeners and crop growers”* were included because they could have been misclassified crop and animal producers.

It is worth noting that the Norwegian names of all these occupational groups (61 and 611–613), used by the interviewers, imply that the study participants are independent owner-farmers. Agricultural workers who are employed by a farming enterprise would be classified as *92* and/or *921 “Agricultural*, *fishery and related laborers”* (n = 28, also includes fishery/related laborers). This latter group includes largely unskilled manual workers (classified under “*9 Elementary occupations”)* who do not own the farm they are working on, and were not included in our study. The full list of occupational categories used in HUNT4 and numbers of participants in each category can be found in the HUNT Databank [66].

No exclusion criteria, for example based on age or current occupational activity, were made at this stage.

### The data merging process

**Fig 1:**
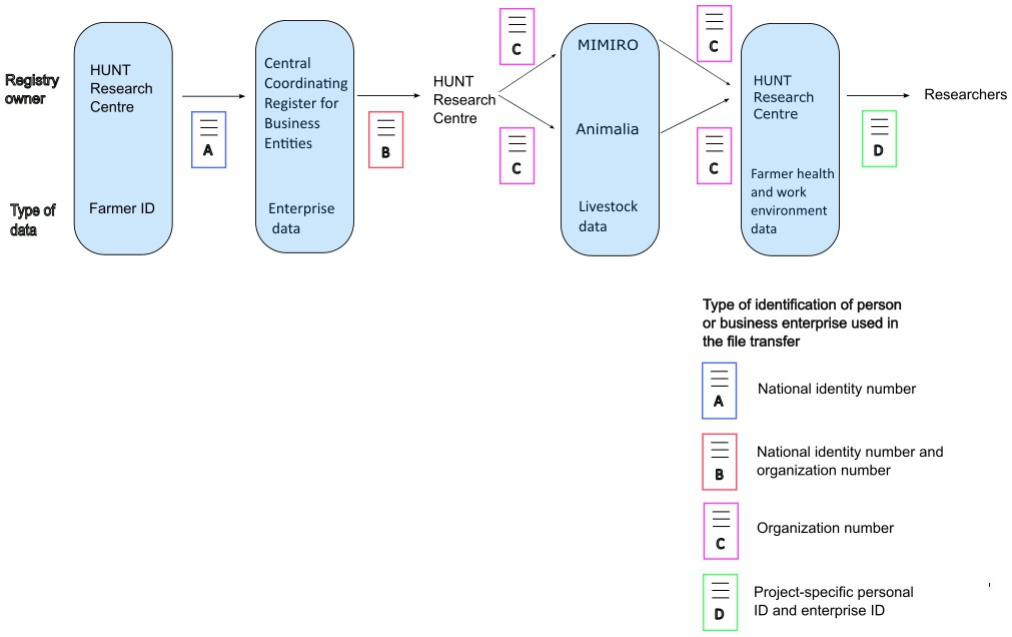
The data merging process.

In the HUNT Study, the 11-digit **Norwegian national identity number** [67] is used as the identifier of the study participants. This national identifity number is not, however, consistently used in the livestock databases. To bridge the gap between the farmer and the enterprise, we used the CCR to obtain the **organization numbers** of enterprises that the HUNT4 farmers had a registered role in.,

The data merging process, including which identification variables (of farmers and/or farming enterprises) were used in each step of the merging process, is shown in Fig 1.

We used CCR data to identify HUNT4 farmers who had an active role in a farming enterprise in the calendar year they participated in HUNT4. In June 2022, the HUNT Research Center sent the list of the national identity numbers of the 2,407 farmers to the Brønnøysund Register Center. Enterprises are, to varying extents, fluid with respect to the precise activities they are engaged in, and the identities and roles of the persons associated with them. To encompass this fluidity, data was retrieved from the CCR at a total of 16 time points, one for each quarter (January 1st, Apri 1stl, August 1st and October 1st) for each of the years 2017–2020—i.e., the years of HUNT 4 data collection plus one subsequent year. This process revealed that 1,715 of the farmers had roles in more than 3,404 unique enterprises, of any kind, at least one time point in the study period (67,849 separate records).

**Fig 2:**
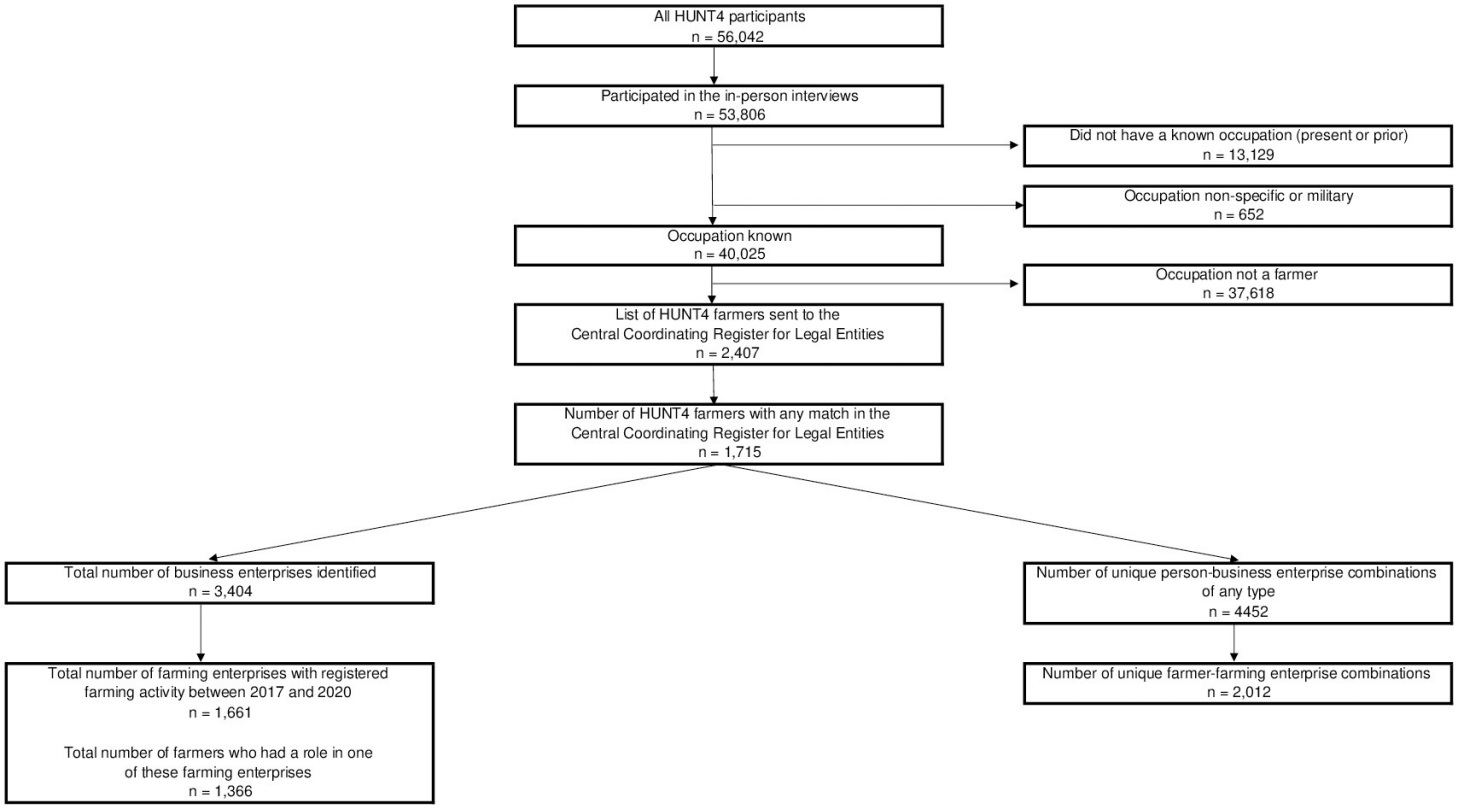
Selection of study participants.

Following the exclusion of enterprises that never registered farming activity at any point between 2017 and 2020, there were 1,661 possible farms to be included in this study. The selection process is illustrated in Fig 2.

A list of the 1,661 organization numbers were sent by HUNT Research Centre to Animalia [68] (NHRS-Beef, NHRS-Sheep, NHRS-Swine and abattoir data) in September 2022, and to MIMIRO [69] (NHRS-Dairy) in December 2022. The files containing livestock data were returned from Animalia and MIMIRO to HUNT Research Centre.

### Data processing

The datafiles from the CCR and the livestock databases were then further assessed and processed by the HUNT Research Center in accordance with the Data Protection Impact Assessment (DPIA). Variables that could potentially be used for direct or indirect identification of study participants were removed or re-categorized.

HUNT Research Centre created project-specific person ID (PID) codes for each study participant, and project-specific enterprise ID codes for each farming enterprise. The national identity numbers and enterprise numbers were then removed, and the pseudonymized files were sent to the researchers in encrypted, password-protected form. Only HUNT Research Center had access to the key.

### Cohort selection criteria

The FarmMERGE cohort was defined as follows: in the year that the farmer participated in HUNT4, the farmer was classified as occupationally active (HUNT4 data), held a formal role at the farm (CCR data), and data on production at the farm was present in at least one of the available livestock database records. Individuals could have a formal role in several farming enterprises, several individuals could have formal roles in the same farming enterprise, and a given enterprise could have more than one type of production.

### Statistical analyses

We used descriptive statistics to describe key characteristics of the farmers selected for inclusion in the FarmMERGE cohort.

The farming enterprises included in the cohort were also described. Furthermore, key characteristics of the farming enterprises were calculated separately based on their reported production form (dairy cattle, beef cattle, all cattle pooled, sheep, swine). Some farms have more than one production form, which resulted in a substantial, and often unknown degree of, overlap between the categories. If there was no membership in any of the herd recording systems, it was not always known which production form the enterprise had. This was particularly relevant for the abattoir data for cattle and swine.

To estimate the overall success of the data merging process, the numbers of unique farming enterprises initially identified in merging process were compared to the mean of the known number of farming enterprises in each production form in the county of Nord-Trøndelag over the period of the HUNT4 data collection (2017–2020). This allowed us to calculate the approximate percentage of HUNT4 participation for each production form. The percentages of enterprises that were selected for inclusion in the cohort were also calculated.

To assess the representativeness of the farming enterprises in the cohort, we compared key statistics on farm size and production to the national and (when available) county averages for 2017. We chose 2017 for comparison, as it was the last year for which separate data was available for Nord-Trøndelag County.

Finally, as one individual could have formal roles in several farming enterprises, and several persons could have formal roles in the same farming enterprise, figures on the number of “farmer-farm combinations” in total, and for each production type and source of data, were calculated.

**Fig 3:**
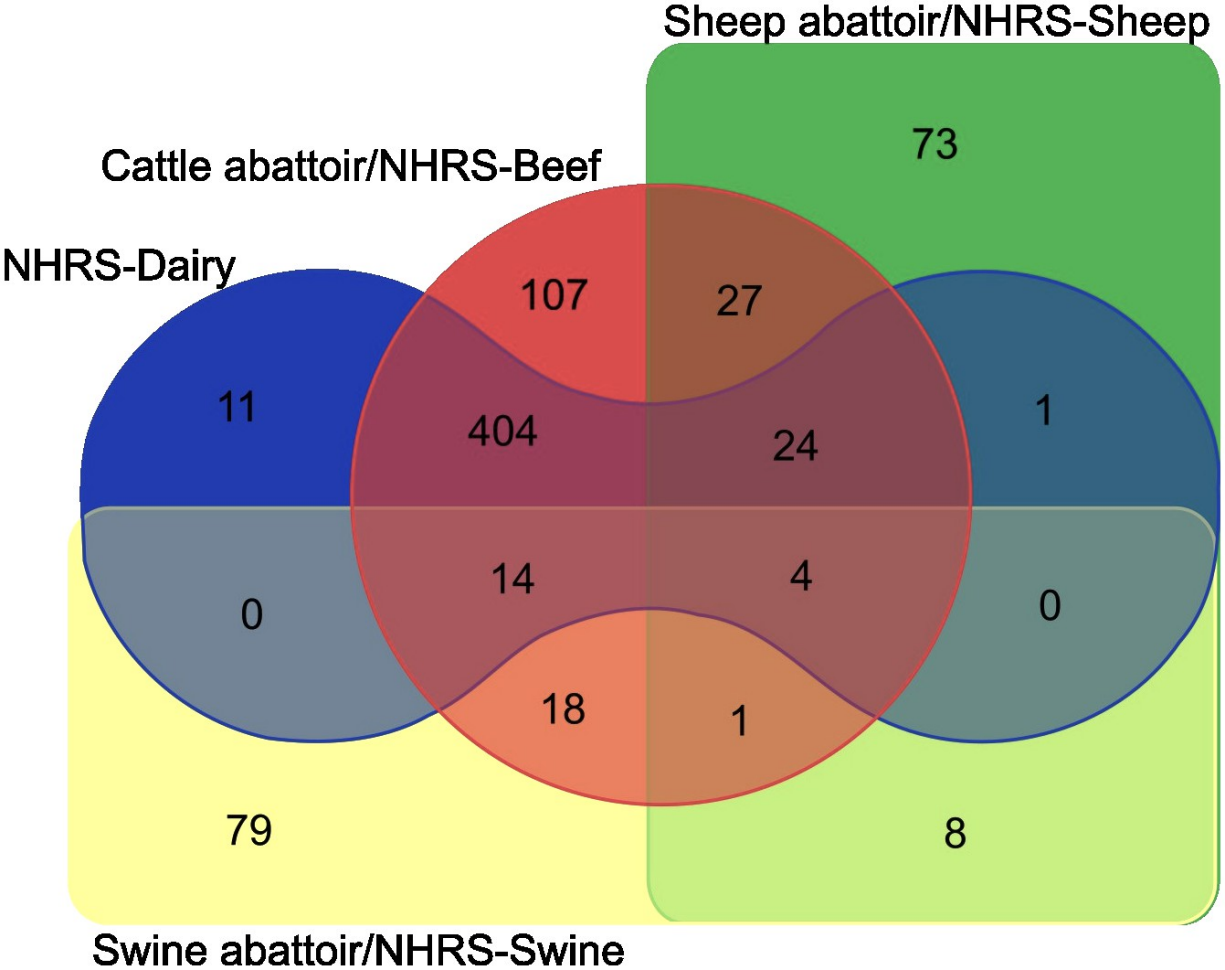
The combinations of production types identified for the 771 farms in the cohort.

The analyses were conducted using R versions 4.2.2 and 4.3.2 [70]. The figures were made using Inkscape v. 1.3.0 (Figs 1 and 3), Microsoft Excel v.2305 (Fig 2), and a free web-based tool available at https://bioinformatics.psb.ugent.be/webtools/Venn/ (Fig 3).

### Research ethics

All HUNT4 participants provided written informed consent, including the consent for their data to be merged with other registries. The informed consent was a part of Q1, and informed consent was repeated when the study participants came to the field stations.

Applications for approval of the current study were sent to the Regional Committee for Ethics in Medical Research (REC Nord) [71] twice, in 2019 and 2021. However, REC Nord considered the project *not* to be medical research as defined by the Act on medical and health research [72], thus not requiring REC approval (reference numbers 34574 and 256719). Thus, the project was approved by the Norwegian Centre for Research Data (NSD, now the Norwegian Agency for Shared Services in Education and Research (SIKT)) [73] in 2020 (reference 923148), and it has a Data Protection Impact Assessment (DPIA) made by NSD. The project also has a data safety plan, which was made using the NSD template and the Nord University data safety measures.

The livestock databases contained routinely collected data. No animals were handled to get data for this project, and the data collection does not classify as animal research according to the Regulation concerning the use of animals in research [74], thus not requiring approval by the Norwegian Food Safety Authority.

## Findings to date

The results of the data merging process and the characteristics of the FarmMERGE cohort are presented first on the ***farmer*** (individual) level, then on the ***farming enterprise*** level, and finally on the ***farmer-farm combination*** level.

### Farmer level

Characteristics of the 816 farmers in the FarmMERGE cohort, for all farmers combined and for farmers within each production system, are shown in Table 1. For comparison purposes, data is also shown on non-farmer participants who reported to be occupationally active at the time of HUNT4 participation, as well as occupationally active farmers not meeting the criteria for inclusion in the FarmMERGE cohort.

**Table 1:** Characteristics of the 816 farmers in the FarmMERGE cohort, compared to other occupationally active HUNT4 participants.

		Farmers in the FarmMERGE cohort										Other occupationally active HUNT4 participants			
		*All farmers in the cohort*		*Cattle farmers*				*Sheep farmers*		*Swine farmers*		*HUNT4 farmers not included in the cohort*		*All non-farmer*, *occupationally active HUNT4 participants*	
				*All cattle farmers* ^a^		*Dairy farmers*									
*Total n*		816		665		513		139		125		743		30915	
*Participated in Q2*		569		472		368		96		85		561		23448	
*Age (years)*	*Range*	22.2–75.3		22.2–74.7		22.2–74.3		26.1–75.3		25.0–70.5		19.2–75.9		19.0–76.0	
	*Median*	52.2		52.4		51.9		50.1		50.1		51.7		47.9	
	*Mean*	51.3		51.1		50.9		50.2		50.5		49.6		46.5	
		*%*	*n*	*%*	*n*	*%*	*n*	*%*	*n*	*%*	*n*	*%*	*n*	*%*	*n*
*Sex (male)*		89.5	816	88.9	665	87.7	513	85.6	139	94.4	125	65.6	743	44.9	30915
*Marital/domestic status*	*Married* ^b^	59.8	816	59.7	665	58.5	513	55.4	139	67.2	125	58.5	743	49.6	30849
	*Lived alone* ^c^	10.7		9.6		10.3		12.2		13.6		12.5		12.3	
*Lifestyle factors*	*Never smoked*	63.5	814	64.0	663	64.4	511	60.0	139	63.2	125	56.1	741	47.8	30812
	*Current daily smoker*	4.9		5.4		5.9		5.8		3.2		5.3		7.4	
	*Possible alcohol dependence* ^d^	11.0	499	11.2	409	10.9	322	9.5	84	10.4	77	12.1	476	11.7	20712
*Highest education level achieved* ^e^	*Compulsory school*	7.0	815	7.4	664	7.4	512	8.6	139	2.4	125	7.4	738	3.8	30808
	*1–2 years of secondary school*	24.5		24.5		24.0		29.5		21.6		22.4		10.9	
	*Secondary school/trade certificate*	45.3		45.0		45.3		40.3		49.6		44.7		37.0	
	*University*	23.2		23		23.2		21.6		26.4		25.4		48.2	
*Self-reported poor or not-so-good health* ^f^		16.5	807	15.9	656	14.2	506	25.4	138	13.7	124	17.9	733	15.1	30608

Many farmers were observed to be active in more than one type of animal production and therefore totals do not equal 100%. If one farmer has e.g. both dairy cattle and sheep, they are included in both columns.

^a^Includes all farmers with cattle, regardless of production type (dairy and/or beef)

^b^As reported in the Norwegian National Population Registry at the time of the HUNT4 invitation. Cohabitants are registered as “unmarried”.

^c^Answered “no” to the question “Do you live with someone”.

^d^Defined by the Cut Down Annoyed Guilty Eye-opener (CAGE) screening instrument, which can be used for detecting alcohol dependency in community settings [75]. The questions relate to ever having experienced any of the following (yes/no): Feeling that one should reduce one’s alcohol consumption, been criticized by others because of one’s alcohol consumption, feeling guilt or discomfort regarding one’s alcohol consumption, and drinking in the morning to “repair”. A cut-off of two or more “*yes*” answers was used. The four CAGE questions were in Q2, and were only asked to participants who answered “*yes*” to the question: *“Do you drink alcohol*?*”*. The reported percentage of alcohol dependency is based on the HUNT4 participants who have answered the CAGE questions only, and is therefore the percentage of those who drink alcohol who report a possible alcohol dependency.

^e^The education question that was asked in HUNT4 was: *“What is the highest level of education that you have completed*?*”* In this table, “compulsory school” equals to 9–10 years of primary and lower secondary school. “Secondary school” is 3 years of secondary school (secondary school diploma) or 3–4 years of vocational school (a certificate of apprenticeship or a trade certificate). “1–2 years of secondary school” could be either vocational or academic, but the level of schooling is presumably not completed, and it is therefore reported separately. “University” includes both “college or university, less than four years” and “college or university, four years or more”.

^f^Self-rated health: Based on the Q1 question “*How is your health at the moment*?”, with the possible response categories of “*poor*”, “*not so good*”, “*good*” or “*very good*”.

The cohort was mostly (89%) male, with the swine industry having the highest percentage of male farmers. For most of the characteristics, the differences between the farmers in different production types were minor. The sheep farmers were possibly the group that differed the most from the rest, in particular in that they had a higher percentage reporting to have “*poor”* or “*not so good”* health, despite their mean age being slightly lower than the mean age of cattle farmers.

### Farming enterprise level

Key herd characteristics of the 771 farms in the FarmMERGE cohort, as well as national herd characteristic means from 2018, are shown in Table 2. The year of 2018 was chosen for comparison because that was the year during which the majority of the farmers in our sample participated in HUNT4 (507 out of the 816 farmers). If there was production data for a farm for more than one year in the 2017–2019 period, we used the data for the year the farmer participated in HUNT4. Compared to the national means, the farms in our sample were similar in most respects. The number of sow equivalents/year and the daily weight gain in lambs were lower than the national mean, whereas a higher proportion of dairy farms in our sample had an Automatic Milking System (AMS) (49.9%), compared to the national average of 24.3%.

**Table 2:** Key characteristics of the 771 farming enterprises in the cohort in the year the associated farmer/s participated in HUNT4, compared to the Norwegian mean herd characteristics in 2018.

Herd characteristics	Data source registry	Herd characteristic value in our cohort^a^ (Mean and 95% confidence intervals)	Number of farms in our cohort from which the herd characteristic mean is calculated	National mean of herd characteristic in 2018^b^
***Cattle***				
Cow equivalents per herd per year *Dairy herds*	NHRS-Dairy	29.3 (27.6, 31.1)	396	27.8
Cow equivalents per herd per year *Beef herds*	NHRS-Beef	21.0 (16.9, 25.1)	39	20.6
Milking system (%AMS)	NRHS-Dairy	49.9% (44.7, 55.0)	373	24.3
Kg milk per cow equivalent per year	NHRS-Dairy	7652 (7505, 7800)	443	7987
Calving interval (months) *Dairy herds*	NHRS-Dairy	12.4 (12.3, 12.5)	434	12.5^c^
Calving interval (months) *Beef herds*	NHRS-Beef	All breeds: 11.7 (10.3, 13.2)	39	12.7
		Heavy breeds: 10.8 (9.0, 12.5)	27	N/A
		Light breeds: 12.2 (10.3, 14.0)	24	N/A
Calf mortality before 6 months^d^ *Dairy herds*	NHRS-Dairy	5.2 (4.7, 5.7)	410	N/A
Calf mortality before 180 days *Beef herds*	NHRS-Beef	All breeds: 5.6 (3.7,7.5)	39	4.10%
		Heavy breeds: 6.1 (3.7,8.4)	27	N/A
		Light breeds: 3.8 (1.4,6.2)	24	NA
Infection level (%)	NHRS-Dairy	19.9 (19.2, 20.6)	410	21
***Sheep (all breeds combined)***				
Number of ewes bred/herd	NHRS-Sheep	127.8 (115.7, 139.9)	39	N/A
Mean number of lambs born/ewe (liveborn and stillborn)	NHRS-Sheep	2.15 (2.06, 2.23)	39	2.04
Average number of lambs slaughtered/year	Abattoir	117.8 (99, 136.6)	138	N/A
Daily growth from birth until autumn/weaning (g/day)	NHRS-Sheep	232.7 (217.3, 248.1)	39	280
***Swine***				
Number of sow equivalents/herd	NHRS-Swine	75.1 (61, 89.1)	27	117
Dead before weaning (%)	NHRS-Swine	12.9 (11.3, 14.4)	27	12.5
Farrowing rate (%)	NHRS-Swine	81.1 (79.7, 82.6)	27	82.7
Daily growth of fattening pigs (g/day)	NHRS-Swine	1033.8 (1016.3, 1051.3)	15	1032

N/A = Not available in the livestock database reports. AMS = Automatic Milking System

^a^If the herd characteristic for a farm was available for more than one year, due to more than one HUNT 4 participant being associated with the farm, only the data for 2018 was used.

^b^National means of members of the herd health system members only. The sources of the figures in this column are, with the exceptions of ^c^ and ^e^:

Dairy cattle: A separate report for 2018 was not available, but historical data from 2018 was available in the 2019 yearly report of the NHRS-Dairy [76].

Beef cattle: Bjørnholt et al.: Storfekjøttkontrollen: Årsmelding 2018 [62].

Sheep: Langaker & Lystad: Sauekontrollen: Årsmelding 2018 [63].

Swine: Løfqvist et al.: Ingris: Årsrapport 2018 [61].

^c^2019 figures. Source: Mikalsen et al (2020) [76].

^d^Number of calves lost before 6 months of age, divided by the number of calf days at risk.

^e^Source: Håvard Nørstebø, personal communication 08.09.23.

The approximate proportion of each type of livestock farming enterprises in Nord-Trøndelag County which we were able to match with at least one HUNT4 participating farmer, at minimum one time point between 2017 and 2019, is shown in Table 3. The estimated percentage of farmers initially identified in our study ranged from 30% of sheep farmers to 63% of dairy farmers. The final FarmMERGE cohort represented approximately 27 to 62% of the estimated enterprises in the county.

**Table 3:** The numbers and estimated percentages of farming enterprises in Nord-Trøndelag County identified in this study, and included in the cohort.

Production form	Mean number of farming enterprises in Nord-Trøndelag County in 2017–2019^a^	Number of farming enterprises initially identified by the merge^b^	Estimated percentage of Nord-Trøndelag County farming enterprises initially identified (%)	Number of farming enterprises included in the FarmMERGE cohort	Estimated percentage of Nord-Trøndelag County farming enterprises included in the cohort (%)
Cattle (total)	1192	638	54%	611	51%
Dairy^c^	744	468	63%	458	62%
Sheep	517	153	30%	138	27%
Swine	283^d^ 134^e^	136^f^ 47^g^	48% 35%	124 39	44% 29%

Percentages are calculated from the estimated total number of active farming enterprises in Nord-Trøndelag County (mean in the 2017–2019 period).

^a^Mean over the 2017–2019 period, calculated from the figures in S1 Table. References are available in S1 Table.

^b^The farms could have registered activity in any or all of the years in the 2017–2019 period.

^c^Beef cattle were not included as a separate category, as the number of beef herds is uncertain as the membership percentage of the Norwegian Beef Herd Recording System (NHRS-Beef) is considerably lower than the membership percentage of NHRS-Dairy.

^d^2017 only (all swine).

^e^Average of 2018–2019 only (breeding swine only).

^f^All herds (abattoir data).

^g^Herds that were known from the Norwegian Swine Herd Recording System (NHRS-Swine) to have breeding swine.

Several combinations of production forms were observed in the FarmMERGE cohort enterprises, as shown in Fig 3, which also illustrates the complexity of the dataset. Absence of a combination does not mean it was not present, only that it was not identified in the merging process described.

### Farmer-farm combination level

The numbers of farmer-farm combinations in the FarmMERGE cohort identified in each registry- are included in Table 4. The number of farmer-farm combinations was higher than the number of unique farms identified, reflecting that for 52 enterprises, there was more than one HUNT4 participant with an official role in the CCR.

**Table 4:** All farmers, farms and farmer-farm combinations in the FarmMERGE cohort.

Species	HUNT4 farmers	Source registry	Farms	Farmer-farm combinations
*All Cattle*		NPRS-Beef	39	39
		NPRS-Dairy	458	513
		Abattoir	599	663
Total	665		611	675
*Sheep*		NPRS-Sheep	39	39
		Abattoir	138	139
Total	139		138	139
*Swine*		NPRS-Swine	39	39
		Abattoir	123	124
Total	125		124	125
**Total (unique)**	**816**		**771**	**835**

Some farms were identified in more than one registry. NHRS-Dairy = Norwegian Dairy Herd Recording System. NHRS-Beef = Norwegian Beef Herd Recording System. NHRS-Sheep = Norwegian Sheep Herd Recording System. NHRS-Swine = Norwegian Swine Herd Recording System.

The number of farmer-farm combinations was limited for data originating from the Herd Recording Systems of beef, sheep, and swine.

### Ongoing activities and future plans

The project has published one paper on the health of farmers [10], which utilized the whole farmer population in HUNT4, not the cohort described in this paper. A corresponding manuscript on work environment is also in preparation. Using the cohort described in this paper, we are currently investigating associations between farmer health exposure variables (both physical and mental health) and selected livestock health/production/welfare outcomes. In the future, we are also planning to do the same for selected aspects of farmer work environment.

## Strengths and limitations

The FarmMERGE data merging was possible due to several distinct assets of Norwegian registries. Firstly, Norway has centrally designated identification numbers, both for individuals and business enterprises, which are used in registries nationwide and can be used to merge data from registries that were originally created for very different purposes. Secondly, a high-quality health study was conducted in a rural area with a significant livestock production. Thirdly, the animal industry registries have either reasonably high participation rates (the herd health control systems) or automatic and/or mandatory registration (slaughter data and veterinary treatment data).

One major strength of the present study is that the human health data comes from a total population-based health study. Study participants may overreport their health problems if they know they have been invited to participate in a study based on their occupation [77]. As all residents of Nord-Trøndelag County were invited to participate in HUNT4, this is less likely to be a source of information bias in our study.

The overall response rate of HUNT4 was 54% [45]. Even though the *true* response rate specifically for farmers in HUNT4 is unknown, our results indicate that that at least dairy farmers may have been more likely to participate in HUNT4 than the general population, and they also indicate that our way of obtaining study participants based on registry identification was successful. The estimated identification rates in other production forms were lower, ranging from 30–54% of the farms in the county. This may indicate real differences in response rates in different groups of farmers, but it may also be caused by our data merging method, especially selecting participants based on farming as their self-reported main occupation. In particular, this decision may have excluded a higher proportion of sheep farmers. Many sheep flocks are small [42, 63], and there is a wide variability between farms in terms of financial performance [78]. As many sheep farmers obtain most of their household income from other sources than sheep farming [29], they are more likely to have an off-farm main occupation than e.g., dairy farmers, who usually work full-time or nearly full-time on the farm [79]. Non-health-related online surveys sent to Norwegian farmers in the recent past have had response rates of 30.7–38.0% [29, 31, 80].

HUNT non-participation studies have found that non-participants had higher mortality, more health problems and more unfavorable health and lifestyle characteristics than participants [45, 81]. It appears likely that the farmers who participated in HUNT4 were healthier and have more favorable lifestyle characteristics than their non-participating colleagues, which may cause bias. This study was not designed to investigate the farmer-livestock association in incidents of severe animal neglect, and any future results from this project should not be extrapolated to include such incidents.

Women are likely to be underrepresented in our cohort. Only 10.5% of the farmers in our cohort were female, compared to 34.4% among HUNT4 farmers not selected for inclusion in the cohort. In 2018, 16% of the farming enterprises (with or without animal production) in Norway had a female listed as the owner/operator, with an even lower proportion (9%) among the largest farms [82]. In a study of currently working HUNT4 participants, 25% of the farmers were female [10]. Our low percentage of female farmers may reflect that female farmers may be less likely to have a formal role in the farming enterprise, and that our method of linking farmer to farming enterprise is less successful in identifying female farmers.

The membership-based Herd Recording Systems have varying membership rates, in some cases below 50% [61, 63]. If the member farms differ from non-member farms, e.g., in terms of farm characteristics, or the farmers’ interest and/or knowledge of agriculture or animal welfare, this could lead to selection bias when using variables from these databases. There may also be differences in the farmer-livestock relationship in large and small herds [83]. It is still worth noting that compared to many other countries, farms in Norway are generally small [84, 85].

We were primarily interested in identifying study participants who had direct responsibility for animal care. We therefore chose a fairly conservative approach when using CCR data to select our cohort, even though it probably decreased our sample size. There are also several possible sources of misclassification, the extent of which are unknown. If the registered formal roles in the CCR do not reflect who actually works on or is responsible for running the farm, this could lead to bias. An alternative approach could have been to merge a list of *all* HUNT4 participants to the CCR, regardless of what they stated as their main occupation. However, this could have resulted in a cohort consisting of a substantial proportion of participants who do not identify as farmers.

Farmer-operators who own their farm are not the only ones who are involved in animal care. We did not include hired farmworkers in our study. Norwegian farms are family-owned, and the farmer-operator (and their family) will often do all, or most of the animal care themself, with often limited hired help [36]. This is also suggested by the nearly 100-fold difference in the number of farmers vs. agricultural/fishery laborers in HUNT4. We considered employed agricultural laborers to be distinctly different from farm owners in several ways (e.g., financial/socioeconomic, less control of work environment, more limited responsibility). Furthermore, with our merging method, we were unable to identify the farms which the 28 agricultural laborers who participated in HUNT worked on, as well as the duration and extent of the responsibility they may have had for a given herd. Therefore, whilst the role of agricultural laborers in livestock care is of interest, their inclusion in this study was precluded by their small number and identification issues, as well as the Norwegian context.

Finally, when data is collected over a period of time, it makes data management and interpretation complex. In our case, this presented with difficulties, particularly because farm production, as well as business registrations and ownerships, are fluid–which may have led to an increased risk of misclassification and inaccurate identification of farming enterprises.

## Conclusions

We have created a cohort of farmers and their farms by merging human, enterprise and livestock databases. Large amounts of data are routinely collected in the livestock industry, and merging already existing databases can be used in future research on stockmanship and the link between the health and welfare of farmers and livestock. The estimated participation rates varied between production forms and was highest for dairy cattle. This is probably a result of our data merging approach, which may not have identified part-time farmers or farmers without a formal role in the farming enterprise. The data merging process was complex and required access to high-quality registries on both humans and livestock, as well as in-depth knowledge on strengths and weaknesses of each database. We have cooperated closely with all the registries to ensure that the data merging would result in a dataset of good quality. Although there are major differences between countries when it comes to access to databases, how they are organized, and how persons and enterprises are identified, the approach we used may be useful in other countries and contexts. We hope that sharing how we merged these databases will be of benefit and inspiration to other researchers, and that our results will benefit farmers, their livestock, and the agricultural industry and society as a whole.

## Supporting information

S1 Table Number of agricultural enterprises with livestock in Nord-Trøndelag County, Norway in 2017–2020. (PDF)

S2 Table Comparison of farm size between Nord-Trøndelag County and Norway as a whole (2017). (PDF)
