# Supplementary material for: Cohort profile: The FarmMERGE project—Merging human and animal databases to investigate the relationship between farmer and livestock health and welfare. The HUNT Study
Source: PLoS One. 2024 Mar 28;19(3):e0301045. doi: 10.1371/journal.pone.0301045 (PMC10977672; doi:10.1371/journal.pone.0301045)
Supplement: S1 Table — (PDF) [file pone.0301045.s001.pdf]

S1 Table. Number of agricultural enterprises with livestock in Nord-Trøndelag County, Norway in 2017-2020

Species/production form	Number of farming enterprises in 2017[a]	Number of farming enterprises in 2018[b]	Number of farming enterprises in 2019[b]	Number of farming enterprises in 2020[b]
Total number of agricultural enterprises with livestock	2,016	1,975	1,911	1,852
Cattle (total)	1,261	1,176	1,140	1,107
Cows (total)	1,183	1,110	1,083	1,040
Dairy cows	817	709	706	665
Beef cows (suckling cows)	449	449	469	474
Sheep (winter-housed/winter-fed)	542	511	499	485
Swine	283[c]	144[d]	124[d]	127[d]
Hens	156	173	127	138
Broilers	104	N/A	N/A	N/A
Dairy goats	4	0	0	5

N/A = not available.

[a]Data source: Statistics Norway, table 03790 [1].

[b]Data source: Statistics Norway, table 06459 [2]. Calculcated by summarizing the individual municipalities that constituted the former Nord-Trøndelag County: Steinkjer, Namsos, Meråker, Stjørdal, Frosta, Levanger, Verdal, Verran (2018-2019), Namdalseid (2018-2019), Snåsa, Lierne, Røyrvik, Namsskogan, Grong, Høylandet, Overhalla, Fosnes (2018-2019), Flatanger, Vikna (2018-2019), Nærøy (2018-2019), Nærøysund (2020) Leka and Inderøy. Years included vary because between 2018 and 2020, several municipalities merged – taking either the old name of one of the municipalities, or a new name [3]. The Nord-Trøndelag municipality of Leksvik was merged with the Sør-Trøndelag county on 1 January 2018 to form the new municipality of Indre Fosen [3]. Consequently, farmers from the former municipality of Leksvik had to be excluded from 2018 onwards, but they are included in the 2017 column.

[c]Swine (total)

[d]Breeding swine (includes gilts, sows young boars and boars that are being used, or are intended to be used in the future, for breeding).
The figures for swine are not comparable between 2017 and 2018-2020 as different measures are used.

References:

1. Statistics Norway. 03790: Jordbruksbedrifter med husdyr per 1. mars, etter husdyrslag (F) 1998 - 2022. Oslo-Kongsvinger: Statistics Norway; 2022 [cited 2022 Dec 1]. Available from: https://www.ssb.no/statbank/table/03790/.
2. Statistics Norway. 06459: Jordbruksbedrifter med utvalde husdyrslag (K) 1969 - 2020. Oslo-Kongsvinger: Statistics Norway; 2021 [cited 2023 May 8]. Available from: https://www.ssb.no/statbank/table/06459/.
3. Trøndelag fylkeskommune. Faktafredag - Hvordan trøndelagskartet har endret seg i årene 2017-2020. Trondheim: Trøndelag fylkeskommune; 2020 [cited 2023 May 8]. Available from: https://www.trondelagfylke.no/vare-tjenester/plan-og-areal/kart-statistikk-og-analyse/nyhetsarkiv-kart-og-statistikk/faktafredag---hvordan-trondelagskartet-har-endrett-seg-i-arene-2017-2020/.
