# Supplementary material for: Cohort profile: The FarmMERGE project—Merging human and animal databases to investigate the relationship between farmer and livestock health and welfare. The HUNT Study
Source: PLoS One. 2024 Mar 28;19(3):e0301045. doi: 10.1371/journal.pone.0301045 (PMC10977672; doi:10.1371/journal.pone.0301045)
Supplement: S2 Table — (PDF) [file pone.0301045.s002.pdf]

S2 Table. Comparison of farm size between Nord-Trøndelag County and Norway as a whole (2017).

**Species/production form**	**Nord-Trøndelag County**		**Norway**	
	**Mean number of animals/farm**	**Mean area/farm (decares)**	**Mean number of animals/farm**	**Mean area/farm (decares)**
Area of arable land		287.1		244.2
Total number of cattle	79.3		63.4	
Dairy cows	30.1		26.9	
Suckling cows	20.4		17.1	
Sheep > 1 year	70.7		65.1	
Milking goats	82.5		113.4	
Breeding swine[a]	85.6		77.4	
Hens	3,857		2,067.9	

[a]Includes gilts, sows, boars and young boars

Sources: Statistics Norway (1-3).

References:

1. Statistics Norway. Holdings, agricultural area and livestock. Oslo-Kongsvinger: Statistics Norway; 2022 [cited 2022 Dec 5]. Available from: https://www.ssb.no/en/jord-skog-jakt-og-fiskeri/jordbruk/statistikk/gardsbruk-jordbruksareal-og-husdyr.
2. Statistics Norway. 04500: Agricultural area per holding (decares) (C) 1969 - 2021. Oslo-Kongsvinger: Statistics Norway; 2022 [cited 2022 Dec 5]. Available from: https://www.ssb.no/en/statbank/table/04500.
3. Statistics Norway. 05985: Number of animals per holding (C) 2000 - 2021. Oslo-Kongsvinger: Statistics Norway; 2022 [cited 2022 Dec 5]. Available from: https://www.ssb.no/en/statbank/table/05985.
